# Mosquito Surveillance for Prevention and Control of Emerging Mosquito-Borne Diseases in Portugal — 2008–2014

**DOI:** 10.3390/ijerph111111583

**Published:** 2014-11-12

**Authors:** Hugo C. Osório, Líbia Zé-Zé, Fátima Amaro, Maria J. Alves

**Affiliations:** Centre for Vectors and Infectious Diseases Research, National Institute of Health Dr. Ricardo Jorge, Av. da Liberdade 5, 2965-575 Águas de Moura, Portugal; E-Mails: libia.zeze@insa.min-saude.pt (L.Z.Z.); fatima.amaro@insa.min-saude.pt (F.A.); m.joao.alves@insa.min-saude.pt (M.J.A.)

**Keywords:** surveillance, mosquito, vector, invasive species, flaviviruses, REVIVE, Portugal

## Abstract

Mosquito surveillance in Europe is essential for early detection of invasive species with public health importance and prevention and control of emerging pathogens. In Portugal, a vector surveillance national program—REVIVE (**RE**de de **VI**gilância de **VE**ctores)—has been operating since 2008 under the custody of Portuguese Ministry of Health. The REVIVE is responsible for the nationwide surveillance of hematophagous arthropods. Surveillance for West Nile virus (WNV) and other flaviviruses in adult mosquitoes is continuously performed. Adult mosquitoes—collected mainly with Centre for Disease Control light traps baited with CO_2_—and larvae were systematically collected from a wide range of habitats in 20 subregions (NUTS III). Around 500,000 mosquitoes were trapped in more than 3,000 trap nights and 3,500 positive larvae surveys, in which 24 species were recorded. The viral activity detected in mosquito populations in these years has been limited to insect specific flaviviruses (ISFs) non-pathogenic to humans. Rather than emergency response, REVIVE allows timely detection of changes in abundance and species diversity providing valuable knowledge to health authorities, which may take control measures of vector populations reducing its impact on public health. This work aims to present the REVIVE operation and to expose data regarding mosquito species composition and detected ISFs.

## 1. Introduction

Rather than relying on an emergency response, mosquito surveillance allows timely detection of changes in abundance and species diversity providing valuable knowledge to health authorities, which may take control measures of vector populations reducing their impact on health. Never as today has the European region been faced with such concern regarding the introduction and dispersion of *Aedes albopictus* and *Ae. aegypti* among other invasive mosquitoes [1,2]. Reports of autochthonous transmission of Chikungunya and Dengue viruses in Italy, France and Croatia [3,4,5,6] and the 2012 Dengue outbreak in Madeira Island [7,8] make obvious the susceptibility to these diseases in areas where the primary mosquito vectors, *Ae. aegypti* or *Ae. albopictus* are present. Moreover, some mosquito species are causing concern, both in posing a risk to public health and causing massive nuisance problems in some areas due to superabundance phenomena [9,10]. The re-emergence of pathogens through native mosquito populations such as West Nile virus (WNV) and malaria has been a major issue in several European regions [11,12,13,14,15,16]. In this context, the need to enhance knowledge about vector species, their distribution, abundance, and role as vectors of disease agents in Portugal, urged the establishment of a national vector surveillance programme—REVIVE (**RE**de de **VI**gilância de **VE**ctores)—in 2008 under the custody of Portuguese Ministry of Health. The REVIVE plan included first the General Directorate of Health (DGS), the five Regional Health Administrations (ARS)—namely Algarve, Alentejo, Lisboa e vale do Tejo, Center and North—and the National Institute of Health Dr. Ricardo Jorge. In 2010, the Institute of Health Administration and Social Services of Madeira Island (IA Saúde) joined REVIVE and started surveillance within the program in seven counties. In 2011, a second protocol was signed between the same partakers, and tick surveillance was also included in the national vector surveillance programme. The main guidelines of REVIVE are (1) surveillance of hematophagous arthropods; (2) seasonal and geographical distribution and abundance of native vector species and early detection of invasive species; (3) identification of pathogenic agents important in public health; (4) surveillance of WNV and other potential flaviviruses in mosquito populations; and (5) alert to the suitability of control measures. The REVIVE has been a key point towards consolidating the establishment of surveillance and vector control at the perimeter of ports and airports according to International Health Regulations [2,17,18,19].

This work aims to present an overview of the REVIVE programme regarding the surveillance of mosquitoes and flaviviruses ongoing in Portugal. The displayed data was produced from mosquito collections performed between 2008 and 2013 on a bi-monthly basis in several Portuguese regions.

## 2. Materials and Methods

### 2.1. Mosquito Collection

Within the framework of arboviruses surveillance programs, the National Institute of Health has been studying the mosquito fauna of several Portuguese provinces to ascertain potential infection risks to the human population [20,21]. The REVIVE programme is based on a strategy of collection ranging from monthly to biweekly or even weekly adult and immature mosquito sampling, which were performed over two to three continuous nights at the same collection site, depending on the particular ability of regional entities to participate in the programme. The periodicity of mosquito sampling is defined in the beginning of every collection season and taken throughout the period, and sampling sites are not changed. The programme has been running since 2008 with field mosquito collection starting in May and ending in October each year. Adult mosquitoes were caught with Centre for Disease Control miniature light traps (CDC traps) baited with CO_2_ and set for a minimum of 12 h periods covering sunset to sunrise. Mosquitoes were inactivated by placing them in a 4 °C refrigerator or killed by freezing and identified under a stereomicroscope, on a chill table, using the identification keys of Ribeiro *et al.* [22] and Schaffner *et al.* [23]. Larvae were commonly collected using a dipper in the same localities and districts and also by occasional collection. Larvae were transported to the laboratory and identified using the same identification keys. 

### 2.2. Ports and Airports

Within the framework of REVIVE, seven ports (Aveiro, Figueira da Foz, Leixões, Portimão, Setúbal, Sines, and Viana do Castelo), one dock (Vila Real de Santo António) and two international airports (Faro and Porto) have been surveyed by ovitraps and CDC light-traps according to International Health Regulations.

### 2.3. Flavivirus Survey

Non engorged females were pooled (a maximum of 50 individuals/pool) by collection date, collection site and species. Pools were grinded in liquid nitrogen and minimal essential medium supplied with 10% FBS, streptomycin (0.1 mg/mL) and fungizone (1 mg/mL), centrifuged 12,000 g for 2 min in and tested for flaviviruses by RT-PCR using the commercial kit SuperScript One-Step RT-PCR (Invitrogen, Carlsbad, CA, USA) after RNA extraction with PureLink RNA Mini Kit (Ambion, Carlsbad, CA, USA). Primers were targeted to a NS5 gene fragment of flaviviruses [24,25]. Amplicons obtained by *Pan-flavi* PCR were purified using JET quick PCR Product Purification Spin kit (Genomed, Löhne, Germany) and sequenced by ABI Prism 3130 Genetic Analyzer (Applied Biosystems, Foster City, CA, USA). The obtained sequences were used to perform basic local alignment searches (BLAST) in GenBank [26]. DNA sequence alignment was performed using Muscle [27] and all phylogenetic analysis was performed in Mega software version 6.06 [28]. Kimura two-parameter model considering the non-uniformity of evolutionary rates among sites using a discrete Gamma distribution with five rate categories and assuming a fraction of sites to be invariable (K2 + G + I; +G = 0.9836 and +I = 0.2225) with the lowest Bayesian Information Criterion was chosen among 24 different nucleotide substitution models [29]. The tree with the highest log likelihood (−3077.01) is presented. The analysis involved 46 sequences and a final dataset of 145 positions (after elimination of all gaps and missing data).

In positive pools by RT-PCR, virus isolation was attempted starting from the remaining part of the homogenates using the C6/36 cell line incubated at 28 ºC [30] and the vertebrate cell line Vero E6.

## 3. Results

**Figure 1:**
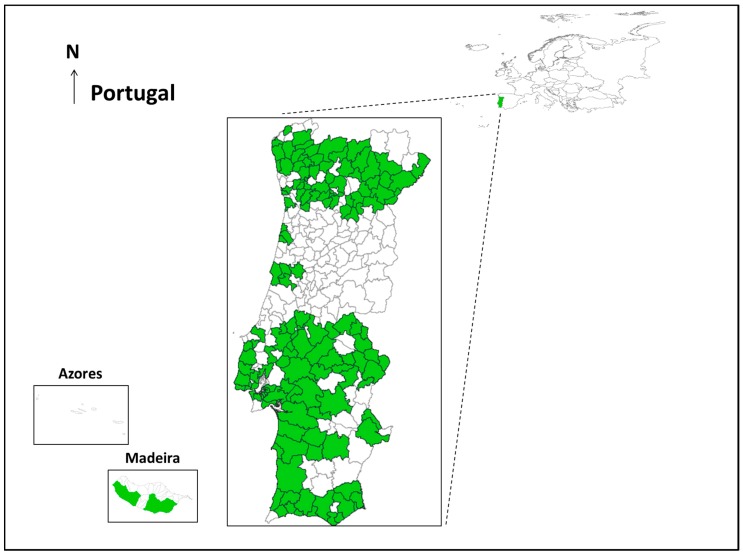
Geographic area under **RE**de de **VI**gilância de **VE**ctores (REVIVE); seven regions, 20 subregions and 151 counties (not to scale).

From 2008–2013 a total of 24 species were recorded in the seven regions (Nomenclature of territorial units for statistics II—NUTS II), 20 subregions (NUTS III) and 151 counties under REVIVE (Figure 1). Between 2008 and 2013, the REVIVE trapped around 500,000 adult mosquitoes, from which 116,808 were identified belonging to 22 species in 3335 trap-nights (Table 1). The most common were *Culex pipiens* (38,941; 33%), followed by *Cx. theileri* (30,845; 26%) and *Ochlerotatus caspius* (30,559; 26%) (Table 2). In the immature collections, 17 species were identified in 3618 positive larvae surveys (Table 1). The three most collected species were *Cx. pipiens* (36,531; 49%), *Culiseta longiareolata* (29,672; 40%) and *Aedes aegypti* (2173; 3%) (Table 2).

A total of 428 trap-nights and 930 immature collections were performed in international ports and airports, resulting in a total collection of 13,984 adult mosquitoes and 2907 immatures (Table 3). In these critical gateways for invasive mosquitoes, only autochthonous mosquitoes were identified in mainland Portugal, mostly from the species *Cs. longiareolata*, *Cx. pipiens* and *Oc. caspius*.

**Table 1:** Summary of collections in the frame of REVIVE.

Collections	Number of	2008	2009	2010	2011	2012	2013	Total
**Adult Collection**	**CDC Trap-night**	267	227	464	725	719	933	3335
	**Adult mosquitoes**	18,310	23,336	25,790	12,739	21,127	15,506	116,808
	**Species number**	15	13	13	22	17	19	22
	**Sub-regions (NUTS III)**	**19**	**11**	**15**	**19**	**20**	**20**	**20**
**Immature Collection**	**Surveyed breeding sites**	143	59	166	518	697	2035	3618
	**Immature mosquitoes**	3338	2125	4562	14,526	25,988	23,850	74,389
	**Species number**	8	7	9	12	10	13	17
	**Sub-regions (NUTS III)**	**14**	**9**	**13**	**19**	**20**	**20**	**20**

**Table 2:** Mosquito species collected in Portugal in the frame of REVIVE.

Species	Larvae	♂ ^1^	♀ ^2^	♂ & ♀ ^3^	Pools ^4^	♀ Pools ^5^	% ♀ Pools ^6^
*Aedes aegypti*	2173	606	195	801	53	725	1.82
*Ae. eatoni*	8		2	2			0.00
*Anopheles algeriensis*		6	437	443	7	278	0.70
*An. claviger*		16	110	126	5	54	0.14
*An. maculipennis* s.l.	295	124	645	769	44	404	1.01
*An. plumbeus*			8	8			0.00
*Coquillettidea richiardii*			75	75	6	55	0.14
*Culiseta annulata*	140	3	237	240	10	62	0.16
*Cs. longiareolata*	29,672	570	439	1009	15	52	0.13
*Culex hortensis*	1578	4	13	17	2	5	0.01
*Cx. impudicus*	120	3		3			0.00
*Cx. laticinctus*	1908	54	25	79			0.00
*Cx. mimeticus*	3	3	8	11	1	6	0.02
*Cx. modestus*	1	1	225	226	6	168	0.42
*Cx. perexiguus*	67	144	1303	1447	79	929	2.33
*Cx. pipiens*	36,531	4727	34,214	38,941	696	19,181	48.11
*Cx. territans*	50						0.00
*Cx. theileri*	1300	648	30,197	30,845	241	8037	20.16
*Cx. torrentium*	359						0.00
*Ochlerotatus berlandi*			9	9	2	17	0.04
*Oc. caspius*	12	1038	29,521	30,559	209	8456	21.21
*Oc. detritus*	172	409	10,553	10,962	40	1409	3.53
*Oc. geniculatus*			55	55	2	21	0.05
*Uranotaenia unguiculata*		114	67	181	1	13	0.03
**Total**	**74,389**	**8470**	**108,338**	**116,808**	**1419**	**39,872**	**100.00**

Notes: ^1^ Number of identified adult male mosquitoes; ^2^ Number of identified adult female mosquitoes; ^3^ Total number of identified adult mosquitoes; ^4^ Number of mosquito pools screened for flaviviruses; ^5^ Number of female mosquitoes screened for flaviviruses in pools; ^6^ Percentage of female mosquitoes screened for flaviviruses in pools.

**Table 3:** Mosquito species identified in the frame of REVIVE in international maritime ports and airports.

Points of Entry	Collections		N		*Ae. aegypti*	*An. maculipennis*		*Cx. hortensis*	*Cx. pipiens*		*Cs. annulata*		*Cs. longiareolata*		*Cx. theileri*	*Oc. caspius*	*Oc. detritus*	
	IM ^1^	AD ^2^	IM	AD	IM	IM	AD	IM	IM	AD	IM	AD	IM	AD	AD	AD	IM	AD
Aveiro	13	62	55	96			1			37			55	14		43		1
Faro-Airport	400	55	725	1934			4		12	599			338	6	139	1025		139
Figueira da Foz	6	63	240	10,206			3		240	863				9	230	9029		72
Funchal (Madeira Island)	1		20		20													
Machico (Madeira Island)	1		7										7					
Porto-Airport	1	41		7						7								
Matosinhos	8	123	111	1378					10	1371		1	101	4	2			
Portimão	55	52	141	308					100	196			41	63	7	40		
Setúbal	228	6		1						1								
Sines	23	15	423	32		1			23	16		3	395	4	4	5	4	
Viana do Castelo	46	11	702	22				44	293	1	74		291	1		20		
Vila Real de Santo António	148		483						201				282					
**Total**	**930**	**428**	**2907**	**13,984**	**20**	**1**		**44**	**879**	**3091**	**74**	**4**	**1510**	**101**	**382**	**10,162**	**4**	**212**

Notes: ^1^ IM—Immatures; ^2^ AD—Adults.

In Madeira, the main concern was the surveillance of the invasive species *Ae. aegypti*. A total of 227 immature collections and 482 trap nights were performed in Madeira since the beginning of 2010, resulting in a collection of 5256 immatures and 1471 adult mosquitoes (Table 4). The most abundant species were *Ae. aegypti* found in five counties of Madeira.

**Figure 2:**
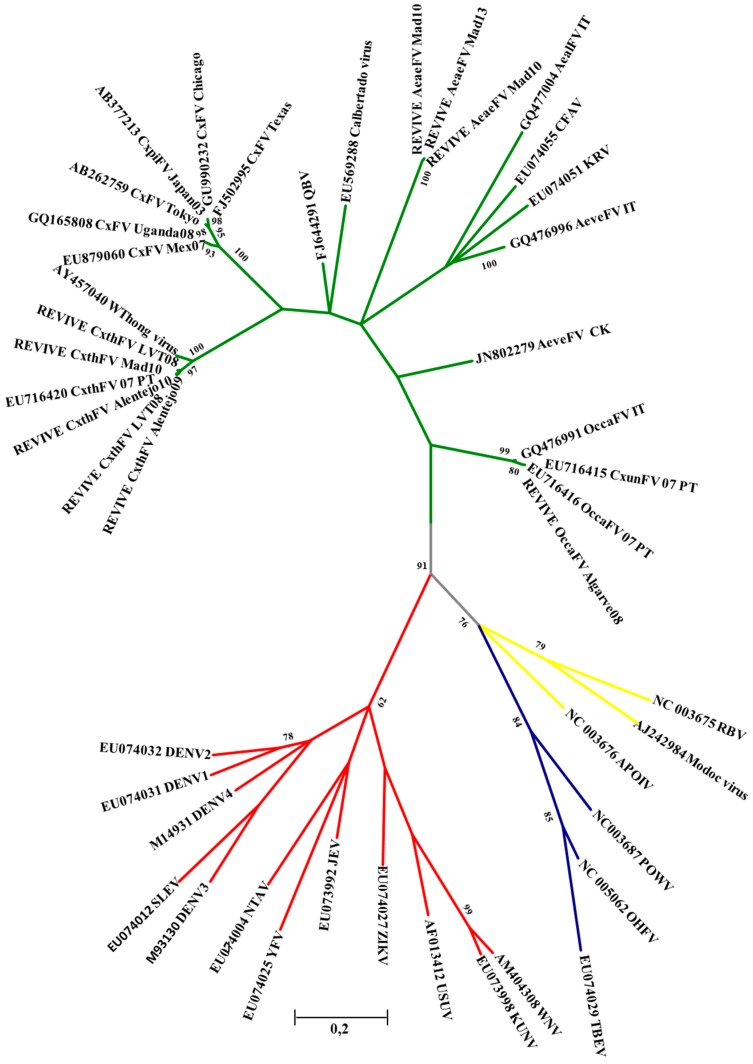
Maximum-likelihood tree obtained from NS5 partial sequences using Kimura two-parameter model of ISFs sequences detected under REVIVE and GenBank sequences of other flaviviruses. Bootstrap values (1000 replicates) above 60% are shown.

Notes: Bar, number of substitutions per site. Branches: green Insect Specific Flavivirus (ISFs); yellow, no-known vector; blue, tick-borne flaviviruses and red, pathogenic mosquito-borne flaviviruses; CK, Czech Republic; IT, Italy; PT, Portugal; LVT, Lisbon and Tagus river region; Mad, Madeira Island.

A total of 39,872 female adult mosquitoes were pooled and screened for flaviviruses surveillance (Table 2 and Table 5). An average of 237 pools and 6645 mosquitoes were processed every year. All pools were negative for the presence of WNV RNA. Insect-Specific Flaviviruses (ISFs) have been detected in *Aedes, Culex* and *Ochlerotatus* mosquitoes. This group of flaviviruses presents a high genetic diversity and, so far, it has only been detected or isolated in mosquitoes, being unable to replicate in vertebrate cells. Within REVIVE three different ISFs have been detected in 36 pools (2.5%): (1) in *Ae. aegypti* from Madeira Island in 2010 (two detections) and 2013 (Genbank representative sequences: HQ676624 and HQ676625); (2) in *Cx. theileri* in Central and Southern regions of Portugal in 2008, 2009 and 2010 and in Madeira Island in 2010 (Culex FV Genbank representative sequences: HQ676619 to HQ676623); and (3) in *Oc. caspius* in 2008 in Algarve (Genbank representative sequence: HQ676618; Table 5 and Figure 2).

**Table 4:** Mosquito species identified in the frame of REVIVE in Madeira Island.

Counties	Collections		N		*Ae. aegypti*		*Ae. eatoni*	*Cs. longiareolata*		*Cx. pipiens*		*Cx. theileri*	
	IM	AD	IM	AD	IM	AD	AD	IM	AD	IM	AD	IM	AD
Calheta	20	1	141	23	136	23				5		20	
Câmara de Lobos	33	123	805	464	715	338	1	82	31	8	88	33	6
Funchal	161	358	4154	984	1295	440	1	261	159	1967	270	161	114
Machico	4		57					57				4	
Ponta do Sol	1		14		12					2		1	
Ribeira Brava	2		8									2	
Santa Cruz	6		77		15			51		11		6	
**Total**	**227**	**482**	**5256**	**1471**	**2173**	**801**	**2**	**451**	**190**	**1993**	**358**	**227**	**120**

**Table 5:** Flaviviruses detected in mosquitoes collected in Portugal in the frame of REVIVE.

Number of	2008	2009	2010	2011	2012	2013	Total
*Pools*	214	164	230	328	251	232	**1419**
♀ *Pools*	6785	5230	5093	7425	7947	7392	**39,872**
*Culex* FV	5	0	3	7	0	0	**15**
*Ochlerotatus* FV	1	1	1	4	0	0	**7**
*Ae. aegypti* FV	0	0	3	7	0	4	**14**

Notes: Genebank sequences representative of ISFs: HQ676619 to HQ676623 (*Culex* FV); HQ676618 (*Ochlerotatus* FV); HQ676624 and HQ676625 (*Ae. aegypti* FV).

## 4. Discussion

Mosquito-borne diseases are (re-) emerging threats to Europe where globalization and environmental changes, together with other interacting drivers such as social and demographic change and health system policy, pose obvious challenges when it comes to vector-borne diseases [18]. Rather than relying on an emergency response, REVIVE allows timely detection of changes in abundance and species diversity providing valuable knowledge to health authorities, the scientific community and entities which may take control measures of vector populations reducing their impact on public health.

Since its inception in 2008, REVIVE has been responsible for the establishment and implementation of guidelines for the surveillance of arthropod vectors of disease with impact on public health. These guidelines were set according to the European Centre for Disease Prevention and Control (ECDC) guidelines for the surveillance of invasive mosquitoes in Europe, in order to be in line at a European level with other countries/areas under surveillance [10]. Methods and information records for the surveillance of invasive mosquitoes in Europe are being followed and since 2010 the REVIVE collaborates with the European Network for Arthropod Vector Surveillance for Human Public Health (VBORNET). In 2014 the REVIVE was ongoing in all points of entry under international regulation (PoE) in Portugal. The primary vector for dengue *Aedes aegypti* is now found in Madeira Island, Portugal, where in 2012 was responsible for the first dengue outbreak within European territory since the 1926–1928 outbreak in Greece [7]. More than 2000 cases and 78 exportation cases into 13 other European countries via travelers departing Madeira were reported [31]. Taking this occurrence as an example, updated information on mosquito species and their geographic distribution is essential more than ever to reduce the impact of vector-borne diseases by providing an opportunity to initiate preventive actions and control methods, such as vector control, prior to the emergence of diseases in human population. According to Ribeiro and Ramos [22], the Portuguese mosquito fauna includes 45 species and subspecies distributed in 15 genera and seven subgenera. Some of the species listed in this publication have a limited distribution, are sporadic or considered only for their potential presence. The data obtained in REVIVE programme about potential vector species composition is in agreement with the work published by Ribeiro with some updates regarding geographical distribution of some species.

One useful output of REVIVE is the generation of abundance and population data on indigenous mosquito species in order to assess the ability of WNV to establish at a regional level. In Portugal, WNV was demonstrated to be circulating in epizootic transmission since 1966/1967 and was first isolated in 1969 from *Anopheles maculipennis* [32,33]. No clinical cases were reported until the summer of 2004 when two tourists acquired WNV in the Southern province of Algarve [34], after which the WNV was isolated from mosquitos sampled from the same region [35]. Every year WNV circulation is detected in birds in Portugal [36]. In 2010 a probable human case of WNV was identified in southern Portugal supporting yearly enzootic circulation of the virus and the risk for human transmission [37].

Insect specific flaviviruses (ISFs) have been increasingly reported from all over the world, and are considered a fourth group within the *Flavivirus* genus unable to replicate in vertebrate cells [38]. Traditionally, flaviviruses are divided into three groups: viruses transmitted by mosquitoes, viruses transmitted by ticks, and viruses with no known vector. The increased number of known ISFs agree with previous studies suggesting a larger number of unsampled taxa in *Flavivirus* genus [39], namely species distantly related to classical arthropod-borne flaviviruses [40,41]. Some studies even indicate that the *Flaviviridae* family or otherwise, the genus *Flavivirus*, may suffer some reorganization in the near future, in a way to better assign this “incoming” species diversity [42]. Furthermore, the presence of DNA sequences similar to ISFs in mosquito genomes from the genera *Aedes* and *Ochlerotatus* also suggests a close evolution between these viruses and their mosquito hosts [43,44,45,46].

The importance of these viruses in nature has not been yet elucidated; however, it is thought that in situations of co-infection they may prevent the transmission of pathogenic virus. Although there are studies supporting this hypothesis [46,47], there are also evidences for positive interactions between WNV and ISFs [48,49].

Preliminary studies in Marim virus, an ISF isolated from a *Oc. caspius* mosquito pool (collected in Algarve in 2007), indicate that in co-infection or superinfection scenarios the replication of WNV seems to be reduced (unpublished data). Marim ISF groups with *Ochlerotatus* FV (Table 5 and Figure 2), and unlike most described ISFs, was detected in different mosquito genera and species (*Cx. univitattus*, *Cx. theileri*, *Cx. pipiens*, *Oc. detritus*, *Oc. caspius*) in Portugal and Greece (*Oc. caspius*). Considering this WNV suppression hypothesis, the presence of naturally infected mosquito populations with ISFs could decrease the risk of human infection in Portugal. However further studies are needed to clarify the interactions of Marim virus on WNV infection, dissemination and transmission.

## 5. Conclusions

The REVIVE represents a successful layout for mosquito surveillance at national level, in which entomologists, virologists, public health professionals, and policy makers have a high degree of cooperation aiming for an effective surveillance program. With seven years of experience, additional challenges include: to increase the surveyed area, mostly in the Central region of the country and also including Azores Islands; to set collection sites in points of entry from Spain, thus increasing the ability for early detection of *Ae. albopictus*; and the cooperation and closer integration with surveillance across national borders at a European level, contributing to a Europe-wide surveillance network. This would greatly assist in data comparison and sharing among European regions.
